# Exact solutions of unsteady Korteweg-de Vries and time regularized long wave equations

**DOI:** 10.1186/s40064-015-0893-y

**Published:** 2015-03-12

**Authors:** S M Rayhanul Islam, Kamruzzaman Khan, M Ali Akbar

**Affiliations:** Department of Mathematics, Pabna University of Science & Technology, Pabna, 6600 Bangladesh; Department of Applied Mathematics, University of Rajshahi, Rajshahi, 6205 Bangladesh

**Keywords:** The exp(−Φ(ξ))-expansion method, The TRLW equation, The KdV equation, NLEEs, Exact solutions

## Abstract

In this paper, we implement the exp(−Φ(ξ))-expansion method to construct the exact traveling wave solutions for nonlinear evolution equations (NLEEs). Here we consider two model equations, namely the Korteweg-de Vries (KdV) equation and the time regularized long wave (TRLW) equation. These equations play significant role in nonlinear sciences. We obtained four types of explicit function solutions, namely hyperbolic, trigonometric, exponential and rational function solutions of the variables in the considered equations. It has shown that the applied method is quite efficient and is practically well suited for the aforementioned problems and so for the other NLEEs those arise in mathematical physics and engineering fields.

**PACS numbers:** 02.30.Jr, 02.70.Wz, 05.45.Yv, 94.05.Fq.

## Introduction

Most of the real world problems are generally modeled by NLEEs. The study of exact traveling wave solutions for NLEEs play an important role in the study of nonlinear physical phenomena. Therefore, finding explicit solutions of physics equations is an important and interesting subject.

In this paper, we consider two NLEEs which have a great importance in mathematical physics. The first one is Korteweg de Vries equation, derived by Diederik Johannes Korteweg together with his PhD student Gustav de Vries, now well known as the KdV equation (Wazwaz 2009), having the simplest form

1 $$ {u}_t+u{u}_x+\delta \kern0.24em {u}_{xxx}=0, $$

where *δ* is a nonzero constant. The term *u*_*t*_ in this equation describes the time evolution of the wave propagating in one direction. Moreover, this equation incorporates two adversary effects: nonlinearity represented by *uu*_*x*_ that accounts for steepening of the wave, and linear dispersion represented by *u*_*xxx*_*that* describes the spreading of the wave. Nonlinearity tends to localize the wave while dispersion spreads it out. The balance between these weak nonlinear steepening and dispersion effect formulate the solitons (Wazwaz 2009). The KdV equation is used to model the disturbance of the surface of shallow water in the presence of solitary waves. The KdV equation is a generic model for the study of weakly nonlinear long waves, incorporating leading order nonlinearity and dispersion (Wazwaz 2009; Marchant and Smyth 1996). Also, it describes surface waves of long wavelength and small amplitude on shallow water (Monro and Parkes 1999, 2000; Zakharov and Faddeev 1971).

And the second equation is the time regularized long wave (TRLW) equation proposed by Joseph and Egri (1977) and Jeffrey (1978), which is one of the alternative form of KdV equation, having the form

2 $$ {u}_t+{u}_x+\alpha\;u\;{u}_x+{u}_{xtt}=0, $$

where *u*, *t* and *x* denote the amplitude, time , and spatial coordinate respectively and *α* is a nonzero constant (Taghizade and Neirameh 2010; Taghizadeha et al. 2012). The TRLW equation shares many of the properties of the KdV equation. Bona and Chen (1999) have shown that the initial value problem for the TRLW equation is well-posed, and that for small-amplitude, long waves, solutions of (2) agree with solutions of (1). The Joseph-Egri (TRLW) equation plays a major role in the study of nonlinear waves since it describes the large number of important physical phenomena, such as shallow water waves and ion-acoustic plasma waves (Hereman 2011).

The exact solutions of NLEEs have been investigated by many authors who are interested in nonlinear physical phenomena which exist in all fields including mathematical physics and engineering fields, such as fluid mechanics, electrodynamics, chemical physics, chemical kinematics, plasma physics, elastic media, optical fibers, solid state physics, biology, and atmospheric and so on.

In recent years, many methods for obtaining explicit traveling and solitary wave solutions of NLEEs have been proposed, such as the extended tanh-method (Abdou 2007; Parkes and Duffy 1996; Yan 2001; Wang and Li 2005a), the F-function expansion method (Wang and Zhou 2003; Wang and Li 2005b), the exp-function expansion method (He and Wu 2006; Khan and Akbar 2014c), the generalized Riccati equation (Wang and Zhang 2007; Wang et al. 2007, Wang et al. 2005), the direct algebra method (Hereman et al. 1986), the complex hyperbolic function method (Zayed et al. 2008), the Modified Simple Equation Method (Khan and Akbar 2014b), the (*G'/G*)*-*expansion Method (Taghizade and Neirameh 2010; Bekir 2008; Khan and Akbar 2014d; Islam et al. 2013; Wang et al. 2008; Zhang et al. 2008) and others. The objective of this paper is to use a new method which is called the exp(−Φ(ξ))-expansion method. This method is firstly proposed by which the traveling wave solutions of non-linear equations are obtained. The main idea of this method is that the traveling wave solutions of non-linear wave equations can be expressed as a polynomial in exp(−Φ(ξ)), where Φ(ξ) satisfies the ordinary differential equation (ODE) *Φ*′(*ξ*) = exp(−*Φ*(*ξ*)) + *μ* exp(*Φ*(*ξ*)) + *λ*, and *ξ* = *x* + *ω t*. The degree of the polynomial can be determined by considering the homogeneous balance between the highest order derivatives and nonlinear terms appearing in the given nonlinear partial differential equation. It will be shown that more traveling wave solutions of many nonlinear evolution equations can be obtained by using the exp(−Φ(ξ))-expansion method.

The rest of the article has been prepared as follows: Description of the exp(−Φ(ξ))-expansion method; applications of exp(−Φ(ξ))-expansion method to find the exact solutions of unsteady Korteweg-de Vries and time regularized long wave equations, graphical representation, and conclusions.

### Description of the exp(−Φ(ξ))-expansion method

In this section we will describe the algorithm of the exp(−Φ(ξ))-expansion method for finding traveling wave solutions of non linear evolution equations. Suppose that a non linear equation in two independent variables *x* and *t* is given by,

3 $$ P\left(u,{u}_t,{u}_x,{u}_{tt},{u}_{xx},{u}_{xt},\dots \dots \dots \dots \right)=0,\kern1em x\in R,\;t>0 $$

where *u*(*ξ*) = *u*(*x*, *t*) is an unknown function, *P* is a polynomial of *u*(*x ,t*) and its partial derivatives in which the highest order derivatives and non linear terms are involved. In the following, we give the main steps of this method (Khan and Akbar 2014a).

Step 1. Combining the independent variables *x* and *t* into one variables *ξ* = *x* ± *ω t*, we suppose that

4 $$ u\left(x,t\right)=u\left(\xi \right)\kern1em \xi =x\pm \omega\;t, $$

where *ω* ∈ *R* − {0} is the velocity of relative wave mode.

The traveling wave transformation Eq. (4) permits us to reduce Eq. (3) to the following ordinary differential equation (ODE),

5 $$ F\left(u,{u}^{\prime },{u}^{{\prime\prime} },\dots \dots \dots \right)=0, $$

where *F* is a polynomial in *u*(*ξ*) and its derivatives, whereas ${u}^{\prime}\left(\xi \right)=\frac{d\;u}{d\xi },\;{u}^{{\prime\prime}}\left(\xi \right)=\frac{d^2u}{d{\xi}^2}$, and so on.

Step 2.We suppose that Eq. (5) has the formal solution

6 $$ u\left(\xi \right)={\displaystyle \sum_{i=0}^n{A}_i}{\left( \exp \left(-\varPhi \left(\xi \right)\right)\right)}^i, $$

where *A*_*i*_, (0 ≤ *i* ≤ *n*) are constants to be determined, such that *A*_*n*_ ≠ 0, and *Φ* = *Φ*(*ξ*) satisfies the following ODE

7 $$ {\varPhi}^{\prime}\left(\xi \right)= \exp \left(-\varPhi \left(\xi \right)\right)+\mu \exp \left(\varPhi \left(\xi \right)\right)+\lambda . $$

Eq. (7) gives the following solutions:

When *λ*^2^ − 4*μ* > 0, *μ* ≠ 0,

8 $$ \varPhi \left(\xi \right)= \ln \left(\frac{-\sqrt{\left({\lambda}^2-4\mu \right)} \tanh \left(\frac{\sqrt{\left({\lambda}^2-4\mu \right)}}{2}\left(\xi +k\right)\right)-\lambda }{2\mu}\right), $$

9 $$ \varPhi \left(\xi \right)= \ln \left(\frac{-\sqrt{\left({\lambda}^2-4\mu \right)} \coth \left(\frac{\sqrt{\left({\lambda}^2-4\mu \right)}}{2}\left(\xi +k\right)\right)-\lambda }{2\mu}\right), $$

When *λ*^2^ − 4*μ* < 0, *μ* ≠ 0,

10 $$ \varPhi \left(\xi \right)= \ln \left(\frac{\sqrt{\left(4\mu -{\lambda}^2\right)} \tan \left(\frac{\sqrt{\left(4\mu -{\lambda}^2\right)}}{2}\left(\xi +k\right)\right)-\lambda }{2\mu}\right), $$

11 $$ \varPhi \left(\xi \right)= \ln \left(\frac{-\sqrt{\left(4\mu -{\lambda}^2\right)} \cot \left(\frac{\sqrt{\left(4\mu -{\lambda}^2\right)}}{2}\left(\xi +k\right)\right)-\lambda }{2\mu}\right), $$

When *λ*^2^ − 4*μ* > 0, *μ* = 0, *λ* ≠ 0,

12 $$ \varPhi \left(\xi \right)=- \ln \left(\frac{\lambda }{ \exp \left(\lambda \left(\xi +k\right)\right)-1}\right), $$

When *λ*^2^ − 4*μ* = 0, *μ* ≠ 0, *λ* ≠ 0,

13 $$ \varPhi \left(\xi \right)= \ln \left(-\frac{2\left(\lambda \left(\xi +k\right)+2\right)}{\lambda^2\left(\xi +k\right)}\right), $$

When *λ*^2^ − 4*μ* = 0, *μ* = 0, *λ* = 0,

14 $$ \varPhi \left(\xi \right)= \ln \left(\xi +k\right), $$

where *k* is an arbitrary constant and *A*_*n*_, *ω*, *λ*, *μ* are constants to be determined later, *A*_*n*_ ≠ 0, the positive integer *n* can be determined by considering the homogeneous balance between the highest order derivatives and the nonlinear terms appearing in Eq. (5).

Step 3. We substitute Eq. (6) into Eq. (5) and then we account the function exp(−Φ(ξ)). As a result of this substitution, we get a polynomial of exp(−Φ(ξ)). We equate all the coefficients of same power of exp(−Φ(ξ)) to zero. This procedure yields a system of algebraic equations whichever can be solved to find *A*_*n*_, *ω*, *λ*, *μ*. Substituting the values of *A*_*n*_, *ω*, *λ*, *μ* into Eq. (6) along with general solutions of Eq. (7) completes the determination of the solution of Eq. (3).

## Applications

### The KdV equation

In this subsection we will apply exp(−Φ(ξ))-expansion method to construct analytical solutions of the KdV equation of the form (1).

The traveling wave transformation equation

15 $$ u=u\left(x,t\right),\xi =x-\omega\;t,u=u\left(\xi \right),u\left(x,t\right)=u\left(\xi \right), $$

transforms Eq. (1) into the following ODE,

16 $$ -\omega\;{u}^{\prime }+u\;{u}^{\prime }+\delta\;{u}^{{\prime\prime\prime} }=0. $$

Integrating Eq. (16) with respect to *ξ* once, yields

17 $$ C-\omega\;u+\frac{u^2}{2}+\delta\;{u}^{{\prime\prime} }=0, $$

where C is integrating constant that can be determine later.

Now taking the homogeneous balance between the highest order derivative *u*″ and the nonlinear term *u*^2^ in Eq. (17), yields

18 $$ u\left(\xi \right)={A}_0+{A}_1\left( \exp \left(-\varPhi \left(\xi \right)\right)\right)+{A}_2{\left( \exp \left(-\varPhi \left(\xi \right)\right)\right)}^2, $$

where *A*_0_, *A*_1_ and *A*_2_ are constants to be determined such that *A*_2_ ≠ 0, while *λ* and *μ* are arbitrary constants.

Substituting *u*, *u*^2^, *u*″ into Eq. (17) and then equating the coefficients of exp(−Φ(ξ)) to zero, we obtain

19 $$ C-\omega {A}_0+\frac{1}{2}{A}_0^2+2\delta {A}_2{\mu}^2+\delta {A}_1\mu \lambda =0. $$

20 $$ \delta {A}_1{\lambda}^2+{A}_0{A}_1-\omega {A}_1+2\delta {A}_1\mu +6\delta {A}_2\mu \lambda =0. $$

21 $$ -\omega {A}_2+\frac{1}{2}{A}_1^2+{A}_0{A}_2+3\delta {A}_1\lambda +8\delta {A}_2\mu +4\delta {A}_2{\lambda}^2=0. $$

22 $$ 10\delta {A}_2\lambda +2\delta {A}_1+{A}_1{A}_2=0. $$

23 $$ \frac{1}{2}{A}_2^2+6\delta {A}_2=0. $$

Solving the above five algebraic equations, yields

$$ C=\frac{1}{2}{\omega}^2-\frac{1}{2}{\delta}^2{\lambda}^4+4{\delta}^2{\lambda}^2\mu -8{\delta}^2{\mu}^2,\;{A}_0=\omega -\delta {\lambda}^2-8\delta \mu $$

$$ {A}_1=-12\delta \lambda,\;{A}_2=-12\delta $$

where *λ* and *μ* are arbitrary constants.

Substituting the values of *C*, *A*_0_, *A*_1_ and *A*_2_ into Eq. (18), yields

24 $$ u\left(\xi \right)=\omega -\delta {\lambda}^2-8\delta \mu -12\delta \lambda \exp \left(-\varPhi \left(\xi \right)\right)-12\delta \exp \left(-2\varPhi \left(\xi \right)\right), $$

where *ξ* = *x* − *ω t*

Now applying Eq. (8) to Eq. (14) into Eq. (24) respectively, we obtain the following seven traveling wave solutions of the KdV equation.

When *λ*^2^ − 4*μ* > 0, *μ* ≠ 0,

$$ \begin{array}{l}{u}_1\left(\xi \right)=\omega -\delta {\lambda}^2-8\delta \mu +\frac{24\delta \lambda \mu }{\sqrt{\lambda^2-4\mu } \tanh \left(\frac{1}{2}\sqrt{\lambda^2-4\mu}\left(x-\omega t+k\right)\right)+\lambda}\hfill \\ {}\kern4em -\frac{48\delta {\mu}^2}{{\left(\sqrt{\lambda^2-4\mu } \tanh \left(\frac{1}{2}\sqrt{\lambda^2-4\mu}\left(x-\omega t+k\right)\right)+\lambda \right)}^2}\hfill \end{array}. $$

$$ \begin{array}{l}{u}_2\left(\xi \right)=\omega -\delta {\lambda}^2-8\delta \mu +\frac{24\delta \lambda \mu }{\sqrt{\lambda^2-4\mu } \coth \left(\frac{1}{2}\sqrt{\lambda^2-4\mu}\left(x-\omega t+k\right)\right)+\lambda}\hfill \\ {}\kern4em -\frac{48\delta {\mu}^2}{{\left(\sqrt{\lambda^2-4\mu } \coth \left(\frac{1}{2}\sqrt{\lambda^2-4\mu}\left(x-\omega t+k\right)\right)+\lambda \right)}^2}\hfill \end{array}. $$

When *λ*^2^ − 4*μ* < 0, *μ* ≠ 0,

$$ \begin{array}{l}{u}_3\left(\xi \right)=\omega -\delta {\lambda}^2-8\delta \mu -\frac{24\delta \lambda \mu }{\left(\sqrt{4\mu -{\lambda}^2} \tan \left(\frac{1}{2}\sqrt{4\mu -{\lambda}^2}\left(x-\omega t+k\right)\right)-\lambda \right)}\hfill \\ {}\kern4em -\frac{48\delta {\mu}^2}{{\left(\sqrt{4\mu -{\lambda}^2} \tan \left(\frac{1}{2}\sqrt{4\mu -{\lambda}^2}\left(x-\omega t+k\right)\right)-\lambda \right)}^2}\hfill \end{array}. $$

$$ \begin{array}{l}{u}_4\left(\xi \right)=\omega -\delta {\lambda}^2-8\delta \mu -\frac{24\delta \lambda \mu }{\left(\sqrt{4\mu -{\lambda}^2} \cot \left(\frac{1}{2}\sqrt{4\mu -{\lambda}^2}\left(x-\omega t+k\right)\right)-\lambda \right)}\hfill \\ {}\kern4em -\frac{48\delta {\mu}^2}{{\left(\sqrt{4\mu -{\lambda}^2} \cot \left(\frac{1}{2}\sqrt{4\mu -{\lambda}^2}\left(x-\omega t+k\right)\right)-\lambda \right)}^2}\hfill \end{array}. $$

When *λ*^2^ − 4*μ* > 0, *μ* = 0, *λ* ≠ 0

$$ {u}_5\left(\xi \right)=\omega -\delta {\lambda}^2-\frac{12\delta {\lambda}^2}{\left( \exp \left(\lambda \left(x-\omega t+k\right)\right)-1\right)}-\frac{12\delta {\lambda}^2}{{\left( \exp \left(\lambda \left(x+\omega t+k\right)\right)-1\right)}^2}. $$

When *λ*^2^ − 4*μ* = 0, *μ* ≠ 0, *λ* ≠ 0,

$$ {u}_6\left(\xi \right)=\omega -3\delta {\lambda}^2+\frac{6\delta {\lambda}^3\left(x-\omega t+k\right)}{\left(\lambda \left(x-\omega t+k\right)+2\right)}-\frac{3\delta {\lambda}^4{\left(x-\omega t+k\right)}^2}{{\left(\lambda \left(x-\omega t+k\right)+2\right)}^2}. $$

When *λ*^2^ − 4*μ* = 0, *μ* = 0, *λ* = 0,

$$ {u}_7\left(\xi \right)=\omega -\frac{12\delta }{{\left(x-\omega t+k\right)}^2}. $$

### The TRLW equation

In this subsection, we will apply the exp(−Φ(ξ))-expansion method to find the exact solutions and then the solitary wave solutions of the TRLW equation of the form (2).

The traveling wave transformation equation is

25 $$ u=u\left(x,t\right),\xi =x+\omega\;t,u=u\left(\xi \right),u\left(x,t\right)=u\left(\xi \right), $$

Eq. (25) transforms Eq. (2) into the following ODE,

26 $$ \left(1+\omega \right)\;{u}^{\prime }+\alpha\;u\;{u}^{\prime }+{\omega}^2{u}^{{\prime\prime\prime} }=0. $$

Integrating with respect to *ξ*, Eq. (26) yields

27 $$ C+\left(1+\omega \right)u+\frac{\alpha }{2}{u}^2+{\omega}^2{u}^{{\prime\prime} }=0, $$

where C is the constant of integration.

Now balancing the highest order derivative *u*″ and non linear term *u*^2^, we obtain *n* = 2.

Hence for *n* = 2, Eq. (6) yields

28 $$ u\left(\xi \right)={A}_0+{A}_1\left( \exp \left(-\varphi \left(\xi \right)\right)\right)+{A}_2{\left( \exp \left(-\varphi \left(\xi \right)\right)\right)}^2, $$

where *A*_0_, *A*_1_ and *A*_2_ are constants to be determined such that *A*_2_ ≠ 0, while *λ* and *μ* are arbitrary constants.

Substituting *u*, *u*^2^, *u*″ into Eq. (27) and then equating the coefficients of exp(−Φ(ξ)) to zero, we obtain

29 $$ C-\omega {A}_0+\frac{1}{2}\alpha {A}_0^2+2{\omega}^2{A}_2{\mu}^2+{A}_0+{\omega}^2{A}_1\mu \lambda =0. $$

30 $$ {\omega}^2{A}_1{\lambda}^2+{A}_1+\alpha {A}_0{A}_1+\omega {A}_1+2{\omega}^2{A}_1\mu +6{\omega}^2{A}_2\mu \lambda =0. $$

31 $$ \omega {A}_2+3{\omega}^2{A}_1\lambda +\frac{1}{2}\alpha {A}_1^2+\alpha {A}_0{A}_2+{A}_2+8{\omega}^2{A}_2\mu +4{\omega}^2{A}_2{\lambda}^2=0. $$

32 $$ 10{\omega}^2{A}_2\lambda +2{\omega}^2{A}_1+\alpha {A}_1{A}_2=0. $$

33 $$ \frac{1}{2}\alpha {A}_2^2+6{\omega}^2{A}_2=0. $$

Solving the above five equations, yields

$$ C=-\frac{1}{2}\frac{-2\omega -{\omega}^2-1+16{\omega}^4{\mu}^2-8{\omega}^4{\lambda}^2\mu +{\omega}^4{\lambda}^4}{\alpha },\kern0.5em {A}_0=-\frac{1+8{\omega}^2\mu +\omega +{\omega}^2{\lambda}^2}{\alpha } $$

$$ {A}_1=-\frac{12{\omega}^2\lambda }{\alpha },\kern0.5em {A}_2=-\frac{12{\omega}^2}{\alpha } $$

where *λ* and *μ* are arbitrary constants.

Now substituting the values of *C*, *A*_0_, *A*_1_ and *A*_2_ into Eq. (28) yields

34 $$ u\left(\xi \right)=-\left(\frac{1+8{\omega}^2\mu +\omega +{\omega}^2{\lambda}^2}{\alpha }+\frac{12{\omega}^2\lambda }{\alpha } \exp \left(-\varPhi \left(\xi \right)\right)+\frac{12{\omega}^2}{\alpha } \exp \left(-2\varPhi \left(\xi \right)\right)\right), $$

where *ξ* = *x* + *ω t*.

Substituting Eq. (8)-Eq. (14) into Eq. (34) respectively, we obtain the following seven traveling wave solutions of the TRLW equation.

When *λ*^2^ − 4*μ* > 0, *μ* ≠ 0,

$$ \begin{array}{l}{u}_1\left(\xi \right)=-\frac{1+8{\omega}^2\mu +\omega +{\omega}^2{\lambda}^2}{\alpha }+\frac{24{\omega}^2\lambda \mu }{\alpha \left(\sqrt{\lambda^2-4\mu } \tanh \left(\frac{1}{2}\sqrt{\lambda^2-4\mu}\left(x+\omega t+k\right)\right)+\lambda \right)}\hfill \\ {}\kern4em -\frac{48{\omega}^2{\mu}^2}{\alpha {\left(\sqrt{\lambda^2-4\mu } \tanh \left(\frac{1}{2}\sqrt{\lambda^2-4\mu}\left(x+\omega t+k\right)\right)+\lambda \right)}^2}\hfill \end{array}. $$

$$ \begin{array}{l}{u}_2\left(\xi \right)=-\frac{1+8{\omega}^2\mu +\omega +{\omega}^2{\lambda}^2}{\alpha }+\frac{24{\omega}^2\lambda \mu }{\alpha \left(\sqrt{\lambda^2-4\mu } \coth \left(\frac{1}{2}\sqrt{\lambda^2-4\mu}\left(x+\omega t+k\right)\right)+\lambda \right)}\hfill \\ {}\kern4em -\frac{48{\omega}^2{\mu}^2}{\alpha {\left(\sqrt{\lambda^2-4\mu } \coth \left(\frac{1}{2}\sqrt{\lambda^2-4\mu}\left(x+\omega t+k\right)\right)+\lambda \right)}^2}\hfill \end{array}. $$

When *λ*^2^ − 4*μ* < 0, *μ* ≠ 0,

$$ \begin{array}{l}{u}_3\left(\xi \right)=-\frac{1+8{\omega}^2\mu +\omega +{\omega}^2{\lambda}^2}{\alpha }-\frac{24{\omega}^2\lambda \mu }{\alpha \left(\sqrt{4\mu -{\lambda}^2} \tan \left(\frac{1}{2}\sqrt{4\mu -{\lambda}^2}\left(x+\omega t+k\right)\right)-\lambda \right)}\hfill \\ {}\kern4em -\frac{48{\omega}^2{\mu}^2}{\alpha {\left(\sqrt{4\mu -{\lambda}^2} \tan \left(\frac{1}{2}\sqrt{4\mu -{\lambda}^2}\left(x+\omega t+k\right)\right)-\lambda \right)}^2}\hfill \end{array}. $$

$$ \begin{array}{l}{u}_4\left(\xi \right)=-\frac{1+8{\omega}^2\mu +\omega +{\omega}^2{\lambda}^2}{\alpha }-\frac{24{\omega}^2\lambda \mu }{\alpha \left(\sqrt{4\mu -{\lambda}^2} \cot \left(\frac{1}{2}\sqrt{4\mu -{\lambda}^2}\left(x+\omega t+k\right)\right)-\lambda \right)}\hfill \\ {}\kern4em -\frac{48{\omega}^2{\mu}^2}{\alpha {\left(\sqrt{4\mu -{\lambda}^2} \cot \left(\frac{1}{2}\sqrt{4\mu -{\lambda}^2}\left(x+\omega t+k\right)\right)-\lambda \right)}^2}\hfill \end{array}. $$

When *λ*^2^ − 4*μ* > 0, *μ* = 0, *λ* ≠ 0,

$$ {u}_5\left(\xi \right)=-\frac{1+\omega +{\omega}^2{\lambda}^2}{\alpha }-\frac{12{\omega}^2{\lambda}^2}{\alpha \left( \exp \left(\lambda \left(x+\omega t+k\right)\right)-1\right)}-\frac{12{\omega}^2{\lambda}^2}{\alpha {\left( \exp \left(\lambda \left(x+\omega t+k\right)\right)-1\right)}^2}. $$

When *λ*^2^ − 4*μ* = 0, *μ* ≠ 0, *λ* ≠ 0,

$$ {u}_6\left(\xi \right)=-\frac{1+\omega +3{\omega}^2{\lambda}^2}{\alpha }+\frac{6{\omega}^2{\lambda}^3\left(x+\omega t+k\right)}{\alpha \left(\lambda \left(x+\omega t+k\right)+2\right)}-\frac{3{\omega}^2{\lambda}^4{\left(x+\omega t+k\right)}^2}{\alpha {\left(\lambda \left(x+\omega t+k\right)+2\right)}^2}. $$

When *λ*^2^ − 4*μ* = 0, *μ* = 0, *λ* = 0,

$$ {u}_7\left(\xi \right)=-\frac{1+\omega }{\alpha }-\frac{12{\omega}^2}{\alpha {\left(x+\omega t+k\right)}^2}. $$

## Graphical representation of some obtained solutions

**Figure 1:**
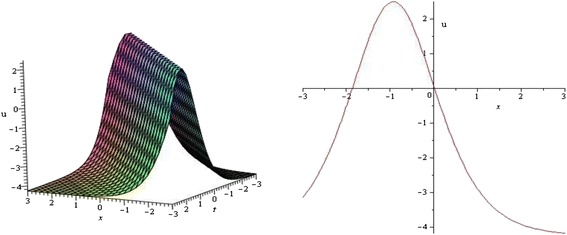
**Bell shaped Soliton profile of KdV equation for** ***λ*** **= 3,** ***μ*** **= 1,** ***k*** **= 0,** ***α*** **= 2,** ***δ*** **= 1 and wave speed** ***ω*** **= 1 within the interval − 3 ≤** ***x*** **,** ***t*** **≤ 3.** (only shows the shape of *u*_1_(*ξ*)), The left figure shows the 3D plot and the right figure shows the 2D plot for *t* = 0.

**Figure 2:**
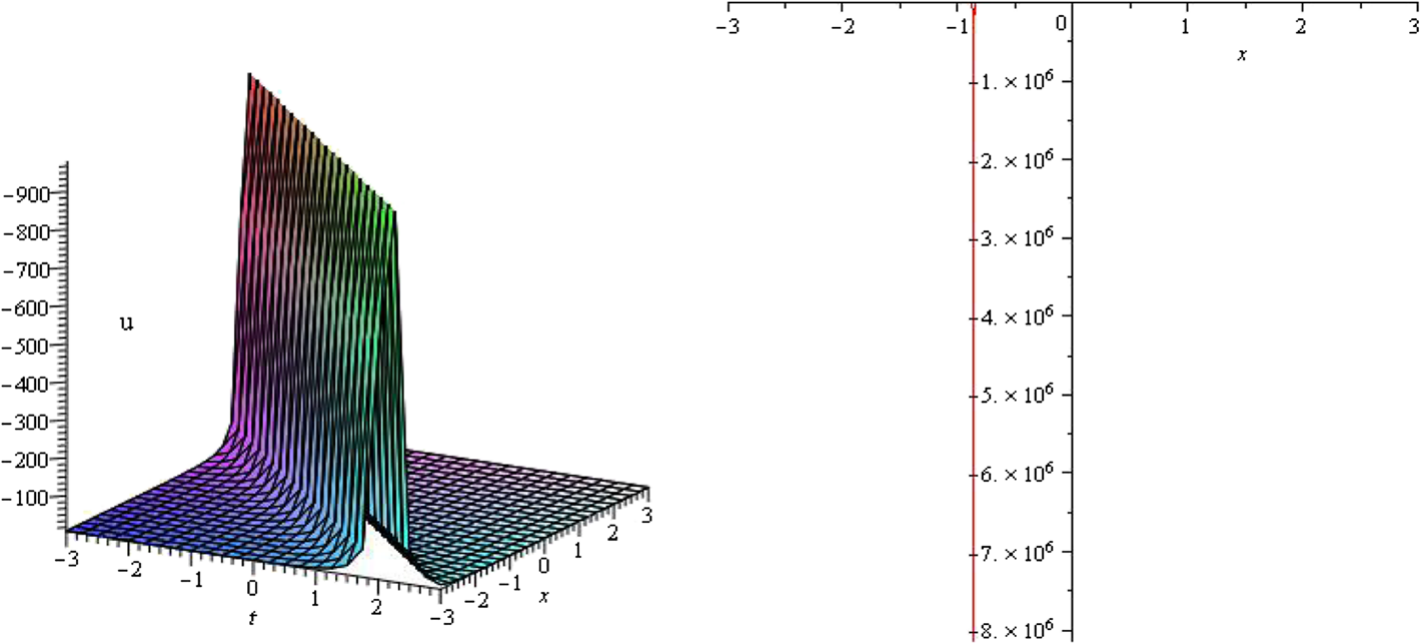
**Singular soliton profile of KdV equation for** ***λ*** **= 3,** ***μ*** **= 1,** ***k*** **= 0,** ***α*** **= 1,** ***δ*** **= 1 and wave speed** ***ω*** **= 2 within the interval − 3 ≤** ***x*** **,** ***t*** **≤ 3.** (only shows the shape of *u*_2_(*ξ*)), The left figure shows the 3D plot and the right figure shows the 2D plot for *t* = 0.

**Figure 3:**
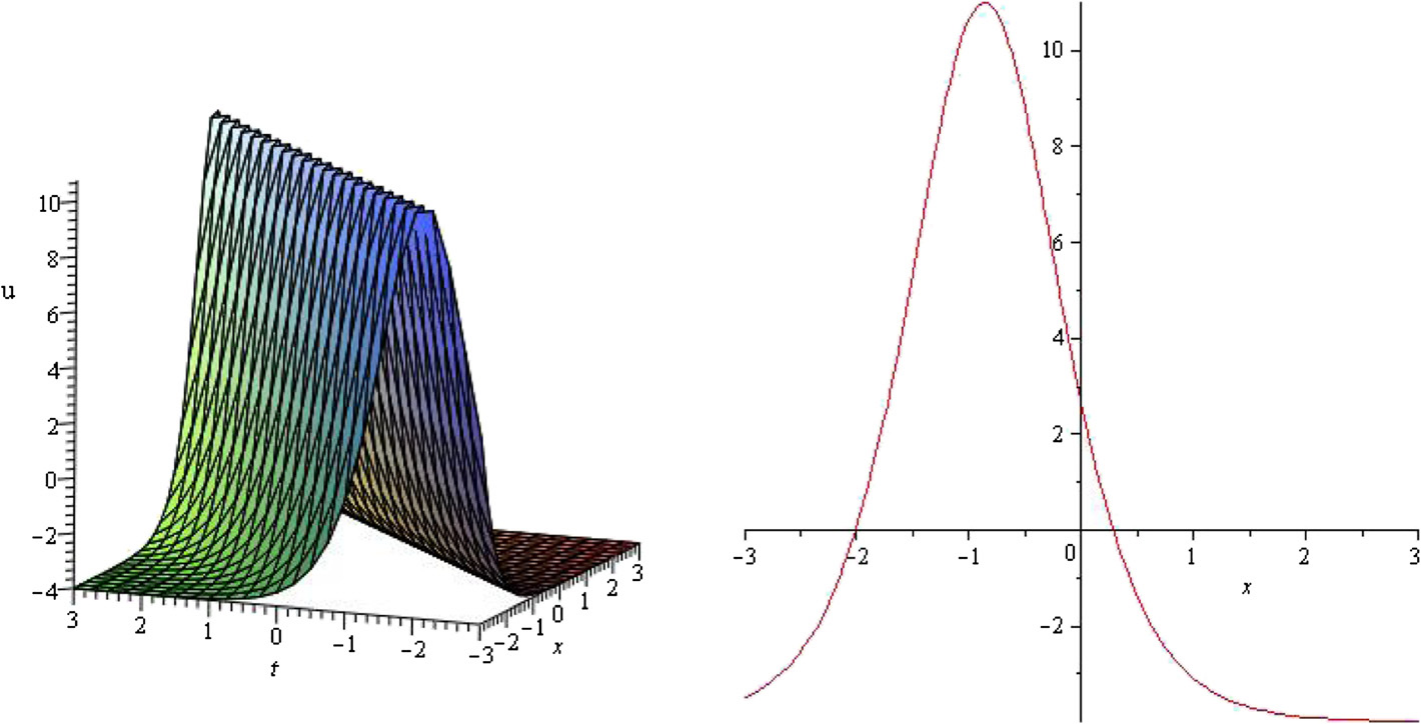
**Bell shaped Soliton profile of TRLW equation for** ***λ*** **= 3,** ***μ*** **= 1,** ***k*** **= 0,** ***α*** **= 1 and wave speed** ***ω*** **= 1 within the interval − 3 ≤** ***x*** **,** ***t*** **≤ 3.** (only shows the shape of *u*_1_(*ξ*)), The left figure shows the 3D plot and the right figure shows the 2D plot for *t* = 0.

**Figure 4:**
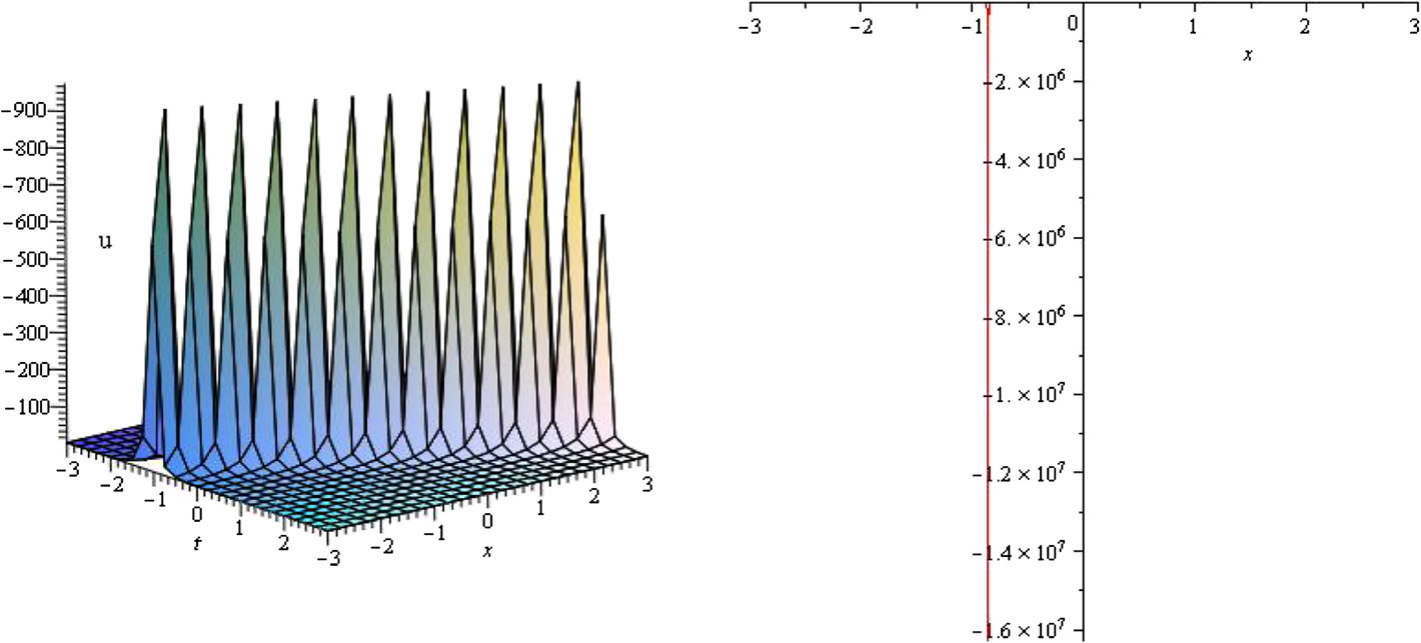
**Singular Soliton profile of TRLW equation for** ***λ*** **= 3,** ***μ*** **= 1,** ***k*** **= 0,** ***α*** **= 1 and wave speed** ***ω*** **= 1 within the interval − 3 ≤** ***x*** **,** ***t*** **≤ 3.** (only shows the shape of *u*_2_(*ξ*)), The left figure shows the 3D plot and the right figure shows the 2D plot for *t* = 0.

Using mathematical software Maple, 2D and 3D plots of some obtained solutions have been shown in Figures 1, 2, 3 and 4 to visualize the underlying mechanism of the original equations.

## Conclusions

In this paper, we have utilized the exp(−*Φ*(*ξ*)) -expansion method to seek exact solutions of the TRLW equation and KdV equation, and found new solutions. The performance of the exp(−*Φ*(*ξ*)) -expansion method is reliable and effective. It can be concluded that this method is very powerful and efficient technique to find the exact solutions for a large class of problems and can be easily extended to all kinds of non linear evolution equations in mathematical physics and engineering.
